# Research Progress and Applications of Multivalent, Multispecific and Modified Nanobodies for Disease Treatment

**DOI:** 10.3389/fimmu.2021.838082

**Published:** 2022-01-18

**Authors:** Jiewen Wang, Guangbo Kang, Haibin Yuan, Xiaocang Cao, He Huang, Ario de Marco

**Affiliations:** ^1^ Department of Biochemical Engineering, School of Chemical Engineering and Technology, Tianjin University, Tianjin, China; ^2^ Frontiers Science Center for Synthetic Biology and Key Laboratory of Systems Bioengineering (Ministry of Education), Tianjin University, Tianjin, China; ^3^ Institute of Shaoxing, Tianjin University, Zhejiang, China; ^4^ Department of Gastroenterology and Hepatology, Tianjin Medical University General Hospital, Tianjin Medical University, Tianjin, China; ^5^ Laboratory for Environmental and Life Sciences, University of Nova Gorica, Nova Gorica, Slovenia

**Keywords:** nanobody multimers, immunomodulation, intrabodies, imaging, nanobody functionalization

## Abstract

Recombinant antibodies such as nanobodies are progressively demonstrating to be a valid alternative to conventional monoclonal antibodies also for clinical applications. Furthermore, they do not solely represent a substitute for monoclonal antibodies but their unique features allow expanding the applications of biotherapeutics and changes the pattern of disease treatment. Nanobodies possess the double advantage of being small and simple to engineer. This combination has promoted extremely diversified approaches to design nanobody-based constructs suitable for particular applications. Both the format geometry possibilities and the functionalization strategies have been widely explored to provide macromolecules with better efficacy with respect to single nanobodies or their combination. Nanobody multimers and nanobody-derived reagents were developed to image and contrast several cancer diseases and have shown their effectiveness in animal models. Their capacity to block more independent signaling pathways simultaneously is considered a critical advantage to avoid tumor resistance, whereas the mass of these multimeric compounds still remains significantly smaller than that of an IgG, enabling deeper penetration in solid tumors. When applied to CAR-T cell therapy, nanobodies can effectively improve the specificity by targeting multiple epitopes and consequently reduce the side effects. This represents a great potential in treating malignant lymphomas, acute myeloid leukemia, acute lymphoblastic leukemia, multiple myeloma and solid tumors. Apart from cancer treatment, multispecific drugs and imaging reagents built with nanobody blocks have demonstrated their value also for detecting and tackling neurodegenerative, autoimmune, metabolic, and infectious diseases and as antidotes for toxins. In particular, multi-paratopic nanobody-based constructs have been developed recently as drugs for passive immunization against SARS-CoV-2 with the goal of impairing variant survival due to resistance to antibodies targeting single epitopes. Given the enormous research activity in the field, it can be expected that more and more multimeric nanobody molecules will undergo late clinical trials in the next future.

Systematic Review Registration

## Introduction

Since the late 1990s, antibodies are largely used in the diagnosis and therapy of neoplastic diseases, including arcitumomab (anti-CEA), capromab (anti-PSMA), and trastuzumab (anti-Her2). At the present, engineered monoclonal IgG antibodies represent the majority of the drugs under development for clinical applications (1, 2). The reasons for their success with respect to small chemical drugs have been thoroughly reviewed (3), as well as are known the negative characteristics of conventional antibodies. They are large molecules with poor penetration capacity in solid tumors, their engineering and site-specific functionalization is difficult to accomplish and leads to the production of heterogeneous populations with variable distribution and efficiency features (4), and they require expensive production and formulation procedures. Recombinant antibody fragments represent a potential solution to most of such drawbacks and, at the same time, preserve the specificity and the sensitivity of full-length antibodies. Historically, Fabs have been the first class of antibody fragments to be successfully exploited (5–8). The possibility of preparing large libraries of unique clones and to pan them against specific antigens allowed then the isolation of antibody fragments in scFv and VHH formats. These miniaturized versions of IgG cannot provide Fc-dependent cellular cytotoxicity and possess no FcRn-dependent prolonged blood circulation but their dimension increases their capacity to penetrate solid tissues and simplifies their humanization and functionalization. Furthermore, they are suitable for the inexpensive production in yeast or bacteria and the design of multivalent or multispecific structures that should improve specificity and apparent binding affinity (9, 10). The possibility to create modular constructs by routine molecular biology techniques is particularly interesting when considering that a single binder should be easily converted into reagents with different characteristics according to the final application. For instance, the clearance of a circulating molecule is strictly dependent on its mass. Consequently, an antibody fragment can be fused to a large partner to increase its persistence in the body for a therapeutic application but kept as small as possible to enable rapid *in vivo* imaging (11). Molecular modifications to extend the serum half-life include conjugation to branched or linear polyethylene glycol (PEG) or fusion with albumin-binding domains, as in the case of ALX-0761 (**Table 1**). Nanobodies, being at the same time the smallest antibody fragments able to preserve the selectivity and sensitivity of the corresponding full-length IgG, maximize the design flexibility for creating immunoreagents customized for specific applications. It is also commonly reported that nanobodies are highly stable. This statement is misleading since lab experience shows that single clones possess very diverging levels of stability and aggregation propensity. However, since library panning usually results in the isolation of a large number of individual clones, in most of the cases it is possible to select at least some candidates with optimal biophysical features for the final applications. As a consequence, “published nanobodies” are usually really stable, despite not being representative of the characteristics of the overall nanobody population.

**Table 1:** Multivalent/bispecific nanobodies that entered clinical trials.

Nanobody	Disease	Target	Structure features	Phase of clinical	Clinical trial
ALX-0061	RA	IL6R	Bivalent albumin-conjugated	Phase II	NCT0251862
ALX-0061	SIE			Phase II	NCT02437890
ATN-103	RA	TNF	Trivalent albumin-conjugated	Phase II	NCT01063803
ALX-0761	Psoriasis	IL17A/IL17F	Trivalent bispecific	Phase II	NCT03384745
			Albumin-conjugated		
M1095	Psoriasis	IL17A/IL17F	Bivalent bispecific	Phase II	NCT03384745
Caplacizumab	TTP	VWF	Bivalent monospecific	Approved	NCT02878603
ALX-0171	RSV	F-protein RSV	Trivalent monospecific	Phase II	NCT02979431
ALX-0651	Healthy volunteers	CXCR4	Bivalent bispecific	Phase I	NCT01374503
BI836880	Solid tumors	Angiopoietin/VEGF	Bivalent bispecific	Phase I	NCT02674152
KN046	Squamous Non small-cell Lung Cancer	PD-L1/TLA4	Tetravalent bispecific	Phase III	NCT04474119
KN046	Advanced HCC	PD-L1/CTLA4	Tetravalent bispecific	Phase I	NCT04601610
KN035	Hepatocellular Carcinoma	PD-L1	Bivalent monospecific	Phase I	NCT03101488
BCMA nanobody CAR-T cells	Relapsed/Refractory Myeloma	CD8/4-1BB	CAR-T	Phase I	NCT03664661
CD7 CAR-T cells infusion	T-lymphoblastic Lymphoma	CD7	CAR-T	Phase I	NCT04004637
CD22 CAR-T cells	B-Cell Lymphoma	CD22	CAR-T	Phase I	NCT03999697
γδT Cell infusion agent	B-cell Leukemia		CAR-T	Early Phase 1	NCT04439721
CD19/CD20 CAR-T cells	B-Cell Lymphoma	CD19/CD20	CAR-T	Phase I	NCT03881761
αPD1-MSLN-CAR T cells	Non-small-cell Lung Cancer	PD-1	CAR-T	Early Phase I	NCT04489862
	Mesothelioma				
αPD1-MSLN-CAR T cells	Colorectal Cancer	PD-1	CAR-T	Phase I	NCT05089266
M6495	Symptomatic Knee Osteoarthritis	ADAMTS-5	Bivalent bispecific	Phase II	NCT03583346

https://clinicaltrials.gov/.

The variety of the applications proposed so far and reported in this work is the confirmation of the nanobody value as reagents for innovative treatments starting from the design of alternative molecular formats. Nanobodies can be grouped together by fusing them to Fc domain or using linkers to construct multimers. Compared with conventional IgG bispecific antibodies, the structure of bispecific nanobodies (BsNb) is simpler to produce and such constructs show excellent solubility and stability. Due to the relevance of the topic, several reviews dealing with general aspects of engineered antibody fragments were published in the last years (12–15). This review will illustrate the research trends in the field of engineered nanobodies designed for disease treatment because this aspect has not yet dealt with systematically. We analyzed recent studies which dealt with the use of BsNbs and other multimeric Nb formats in cancer, immune disease and anti-infective therapy to explore the peculiar characteristics of such macromolecules.

## Nanobody Applications in Cancer Research

Significant progresses with nanobody immunoreagents have been achieved in the fields of α-particle radiation and photodynamic therapy as well as in *in vivo* imaging (16). Despite no nanobody has been yet approved for cancer treatment (**Table 1**), nanobody-based multivalent, multispecific and modified constructs have been tested in a multiplicity of cancer applications with therapeutic potential (17, 18) (**Table 2**) and their recombinant nature enables the production of constructs with a wide range of biodistribution and clearance patterns that optimally fit to different applications (43).

**Table 2:** Multivalent/bispecific nanobodies proposed for cancer therapy.

Nanobody	Disease	Target	Structure features	Year	Reference
MaAbNA	Breast cancer	HER2/EGFR	Bivalent bispecific	2015	(19)
ENb-TRAIL	Lioblastoma	EGFR/DR	Bivalent bispecific	2017	(20)
dhuVHH6-PE38	Acute lymphoblastic leukemia	CD7	Bivalent monospecific	2017	(21)
nanoCAR	B cell leukemia	HER2/CD20	Bivalent bispecific	2018	(22)
7D12-5GS-6H4	Cancer immunotherapeutic	EGFR/Vγ9Vδ2-T	Bivalent bispecific	2018	(23)
α-EGFR-EGFR TM	EGFR^+^ tumor	EGFR	Bivalent monospecific	2018	(24)
RR2-H-RR4	Breast cancer	Her2 epitopes	Bivalent bispecific	2018	(25)
NB-hcAb	Multiple myeloma	CD38	Bivalent monospecific	2018	(26)
Muc1-Bi-2	Ovarian cancer	Muc1/CD16a	Bivalent bispecific	2018	(27)
BiNb	Angiogenesis	VEGF	Bivalent monospecific	2019	(28)
bsVHH	Chronic lymphocytic leukemia	CD1d/Vγ9Vδ2-T	Bivalent bispecific	2019	(29)
BiSS	Colorectal cancer	CEA/CD16a	Bivalent bispecific	2020	(30)
Biss CAR	Acute myeloid leukemia	CD13/TIM3	Tetravalent bispecific	2020	(31)
CD47/CD20 BsAb	Acute myeloid leukemia	CD47/CD20	Tetravalent bispecific	2020	(32)
Bi2	EGFR^+^ tumor	EGFR/FP	Bivalent bispecific	2020	(33)
bi-Nb	Angiogenesis	PLGF	Bivalent monospecific	2020	(34)
NbEGFR-HSA-CD16	EGFR^+^ tumor	EGFR/HAS/CD16	Trivalent tri-specific	2021	(35)
CAM1615HER2	Breast cancer	CD16/HER2/IL15	Bivalent bispecific	2021	(36)
			Antibody-cytokine fusion protein		
S7 ADC	EGFR+ tumor	EGFR	Tetravalent monospecific	2021	(37)
multivalent PD-L1/TIGIT BsAb	Colon cancer	PD-L1/TIGIT	Multivalent bispecific	2021	(38)
C21-7D12/7D12-C21	Colorectal cancer	EGFR/CD16	Bivalent bispecific	2021	(39)
48-(G4S)1-32/32-(G4S)1-48	Leukemia	EGFR/CD16	Bivalent bispecific	2021	(40)
Bispecific Nb CAR	Lymphoma	CD19/CD20	Bivalent bispecific	2021	(41)
11A4-ABD-AF	Breast cancer	HER2/HSA	Bivalent bispecific	2021	(42)

### Imaging

Nanobodies are extremely promising imaging reagents in different clinical applications such as fluorescence-guided surgery (44), positron emission tomography (PET) and ingle-photon emission computed tomography (SPECT) (45, 46).

In cancer *in vivo* imaging, nanobodies have been successfully used to specifically deliver radionucleotides to tumor cells, to biomarkers of the tumor microenvironment and to monitor immune infiltration in animal models and clinical trials (16). The major drawback of the conventional PET protocol that exploits the preferential accumulation of glucose in tumors to deliver radionucleotide-labeled glucose analogues to the cancerous site is that organs such brain or heart, which have high glucose consumption, are difficult to imagine because of the elevated background signal. Antibodies complexed to radionuclides and specific for tumor surface biomarkers represent a rational alternative for selective targeting but the large dimension of IgGs impairs the fast clearance of the unbound fraction. IgGs persist for a couple of days in the vessels determining a diffuse background that prevents high resolution imaging in the first 24-48 hour after administration. However, waiting for days between radionucleotide injection and imaging is impracticable in the clinical organization. Therefore, the optimal solution appears using antibody fragments such nanobodies that are cleared in 15 min by kidney filtration but still provide excellent target accumulation, effective tumor penetration and improved tumor-to-background signal with respect to labeled glucose (47, 48). This approach brings a further advantage: since the imaging can be performed within one hour after the reagent injection, it is possible to use the short half-life positron-emitting nuclides (18F, 68Ga or 89Zr) for PET and the γ-emitting nuclide (99mTc) for SPECT to diminish unnecessary patient irradiation (17). Given the simple engineering of nanobodies, they can be simply expressed as bivalent molecules that still preserve low mass but have increased avidity. For instance, with respect to the monovalent α-EGFR construct, its corresponding bivalent format resulted in higher accumulation at the tumor site and improved PET imaging (24).

### B-Cell Lymphomas/Leukemias

**Figure 1:**
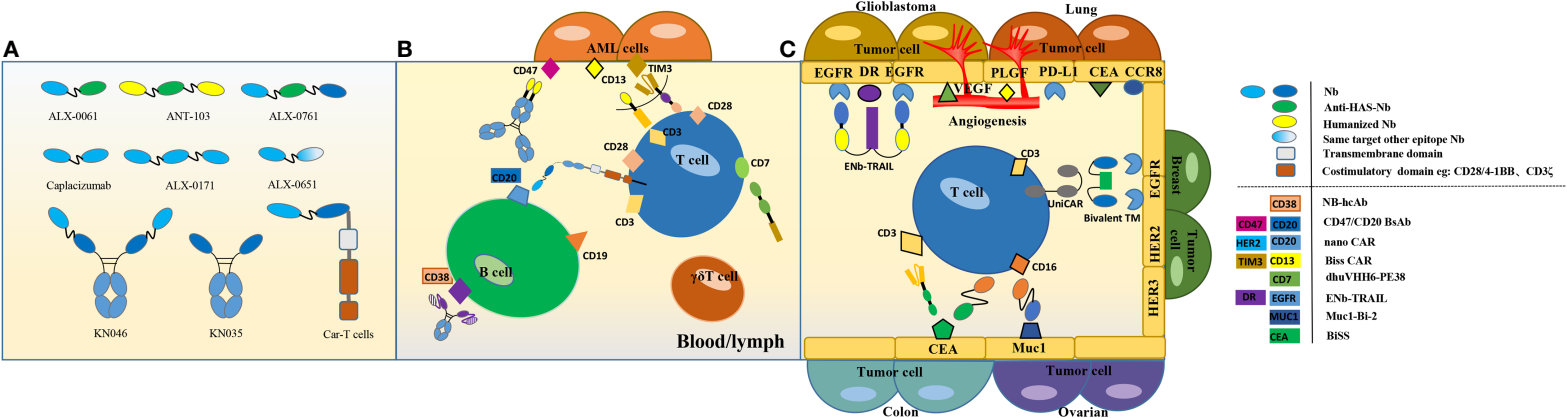
Formats and strategies for multivalent/multispecific nanobodies. **(A)** Formats of multivalent mono/bispecific nanobodies that entered in clinical trials. **(B)** Multivalent mono/bispecific nanobodies applications for blood/lymph cancer therapy. Several CAR-T cell therapies are based on nanobodies and have shown promising effects, for instance in B cell lymphoma. The most commonly targeted receptors on B- and T-cells are CD19, CD20 and CD3, respectively (52). **(C)** Multivalent/bispecific nanobody applications for solid cancer therapy target surface biomarkers of epithelial cancer cells. Multi-functional nanobody structures target multiple epitopes or antigen combinations, resulting in synergistic therapeutic effects for tumors that do not respond to single-target antagonists (53).

B cell lymphoma and leukemia are the most common subtypes of malignant lymphomas and represent 80-85% of non-Hodgkin lymphomas (NHLs). About 20%-40% of B-cell lymphoma/leukemia patients die due to relapse after Rimximab treatment (49). CAR-T cell therapy has shown potential efficacy in the treatment of B cell leukemias and lymphomas and the contemporary targeting of multiple antigen epitopes has been envisaged to overcome the emergence of single antigen-resistant leukemic cells and avoid immune escape (50, 51) (**Figure 1**). Nanobodies targeting CD19, CD20, CD30 and CD22 (52) have been successfully used for CAR applications.

De Munter et al. (22) reported the generation of a bispecific CAR comprising two nanobodies specific for CD20 and HER2, respectively. T cells expressing the bispecific nanoCAR were able to kill tumor cells over-expressing CD20, HER2, or both antigens. Since the individualized manufacturing process of (nano)CARs is costly, the attention moved to donor-derived γδT cells to use as a CAR backbone because γδT cells lack allogenicity and are known to mediate natural anti-tumor responses (54). De Bruin et al. constructed a bispecific nanobody-based structure that targets Vγ9Vδ2-T cells and EGFR for cancer immunotherapeutic therapy (23). Biotech companies such as PersonGen BioTherapeutics developed nanobody-based CAR-γδT cells to treat B-cell leukemia. A clinical trial is currently under way to test the safety and effectiveness of donor γδT cell infusion to prevent relapsed/refractory leukemia rescue relapse after allogeneic hematopoietic stem cell transplantation. Moreover, Vγ9Vδ2-T cells have become a novel potential immunotherapeutic for Chronic Lymphocytic Leukemia (CLL) due to their capacity to be triggered by phosphoantigens which are overproduced by CLL. A nanobody-based CD1d-specific Vγ9Vδ2-T cell engager was generated to induce robust activation and degranulation of Vg9Vd2-T cells and consequent lysis of autologous leukemic cells (29).

### Acute Myeloid Leukemia (AML)

Acute myeloid leukemia (AML) includes all acute non-lymphocytic leukemia. It is related to the insurgency of pluripotent stem cells or slightly differentiated progenitor cell nuclear type mutations. A Sequentially Tumor-Selected Antibody and Antigen Retrieval (STAR) system (31) was developed for screening multiple nanobodies that specifically target AML cells. Nanobodies were used to enhance the binding efficacy of CAR-T cells to AML cells. To this aim, the anti-CD13 nanobody Nb157 was isolated and used to target CD13+ AML cells. Further, bispecific CAR-T cells targeting CD13 and TIM3 were designed to eradicate patient-derived AML and to promote decreased toxicity to human bone marrow stem cells and peripheral myeloid cells in mouse models (**Figure 1**).

CD47 is overexpressed in gastric, ovarian and colon cancer, as well as in AML (55) and an anti-CD47 nanobody (HuNb1-IgG4) with high affinity and specificity (32) effectively empowered macrophage-mediated phagocytosis of tumor cells *in vitro*. *In vivo* it showed potent anti-ovarian cancer and anti-lymphoma activity and its efficacy was further increased when it was combined with rituximab to build a bispecific antibody for the simultaneous targeting of CD47 and CD20.

### Acute Lymphoblastic Leukemia (ALL)

**Figure 2:**
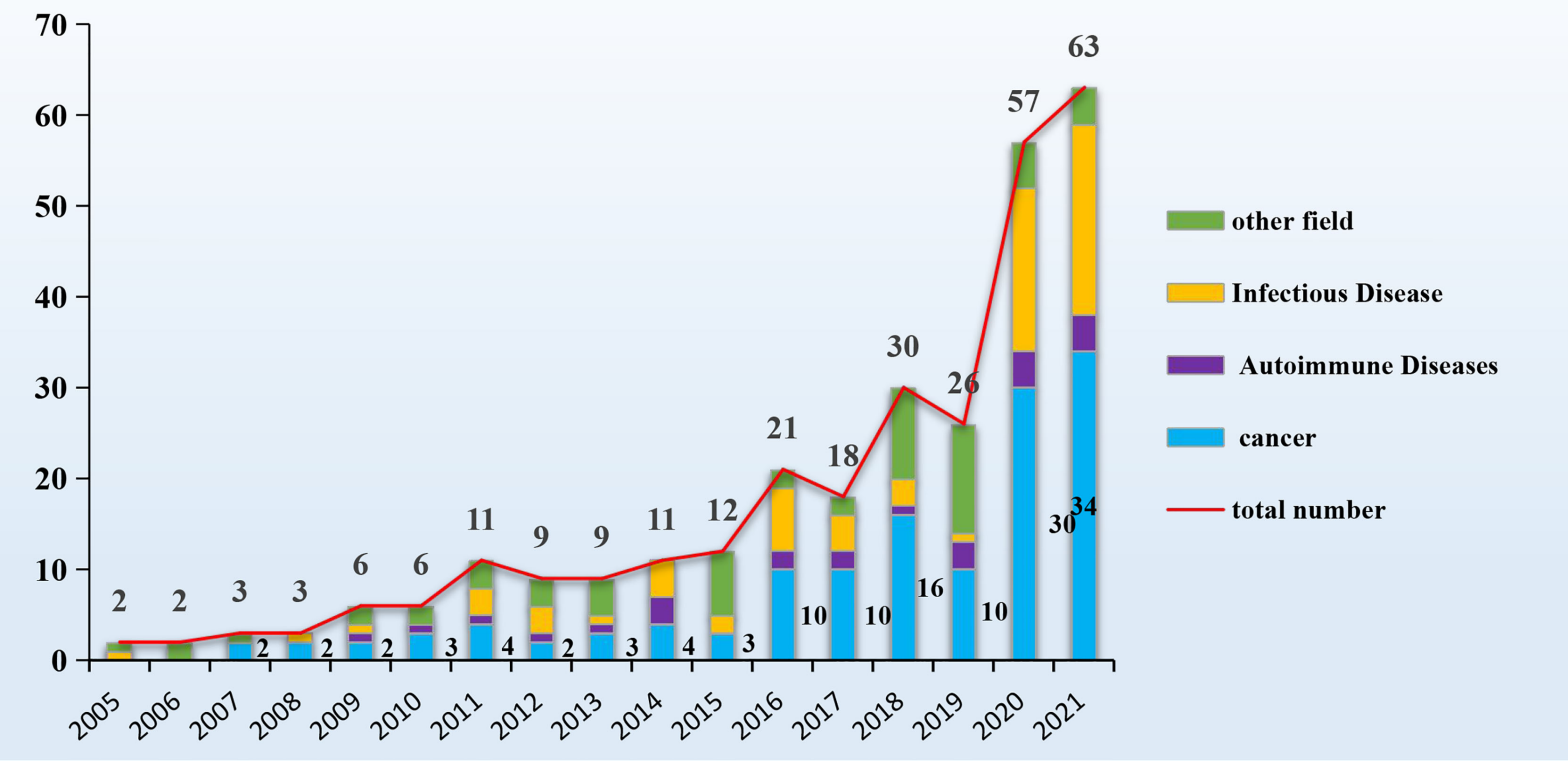
Timeline of conceptual and technical innovations contributing to the development of the multi-function nanobody landscape. The *Camelidae* “heavy-chain-only antibodies” were first reported by Hamers-Casterman et al. (7) in 1993 and the first bispecific antibody (Catumaxomab) was approved by EMA only in 2009 (56). In 2014, the first bispecific scFv (Blinatumomab) was approved by FDA (57) and the same agency approved, the first bivalent nanobody drug (Caplacizumab) for treatment of acquired thrombotic thrombocytopenic purpura (TTP) in 2019 (58).

Acute lymphoblastic leukemia is a highly invasive type of blood cancer. Blinatumomab, which was approved by FDA in late 2014 for the treatment of Fischer-negative precursor ALL (**Figure 2**), is a bispecific antibody based on the BiTE technology and employing two scFvs. One targets the CD19 antigen on the surface of tumor cells, whereas the other targets the CD3 receptor on the surface of cytotoxic T lymphocytes (57). Nanobodies were used in alternative configurations to contrast ALL. CD7 is a convenient ALL biomarker of T-cells (59) because it is rapidly endocytosed once complexed by antibodies and therefore can be exploited for biotherapeutic uptake. A set of humanized anti-CD7 nanobodies was characterized. With respect to the monovalent version, bivalent formats fused to a truncated derivative of *Pseudomonas* exotoxin A showed significant higher cytotoxicity (60, 61). The fusion dhuVHH6-PE38 was used *in vivo* in NOD-Prkdc^em26^IL2rg^em26^Nju (NGG) mouse model and significantly extended the survival of the animals. The same nanobody was successfully used to create CAR constructs which significantly inhibited disease progression in xenograft mouse models of T-ALL primary tumor cells (21).

### Multiple Myeloma

Multiple myeloma is a neoplastic plasma-cell disorder that arises from an asymptomatic premalignant proliferation of monoclonal plasma cells derived from post–germinal-center B cells. CD38 is considered as a biomarker overexpressed in multiple myeloma and the anti-CD38 monoclonal antibody daratumumab demonstrated its high therapeutic efficacy (62).

Schütze et al. isolated a set of nanobodies that recognized three different non-overlapping epitopes of CD38 extracellular domain and then prepared biparatopic constructs by fusing two nanobodies, specific for alternative epitopes, to human IgG1 Fc-domain (26). These constructs outscored both the monoclonal daratumumab and bivalent constructs sharing the same geometry by displaying two identical nanobodies when compared for their capacity of mediating complement-dependent cytotoxicity toward CD38-expressing myeloma cells.

B cell maturation antigen is expressed extensively in malignant plasma cells, seems to be involved in their proliferation and survival and is regarded as a target for CAR-T therapy, despite the possible side effects (63, 64). Nanobody-based CAR-T molecules targeting such receptor are under development in biotech companies but no scientific publication confirms their efficacy.

### Angiogenesis

Tumors require active angiogenesis for securing the energy necessary for their growth and the factors regulating this process become potential drug targets. Vascular endothelial growth factor (VEGF) plays a critical role in the angiogenesis (65) and the high-affinity anti-VEGFR2 nanobody (3VGR19) (28) was tested for its capacity in inhibiting proliferation, tube formation, and migration of human endothelial cells. When built into a bivalent format using the hinge region of llama IgG2c, its half-life *in vivo* in a C57BL/6 mice model was almost doubled and its inhibitory activity was significantly higher than those obtained using the monovalent nanobody.

Placental growth factor (PlGF) is a structurally related member belonging to the same superfamily of VEGF (66) and might be involved in pathogenic angiogenesis, probably by recruiting myeloid progenitors (67). Nikooharf et al. (34) developed a bivalent anti-PLGF nanobody to test as suppressor of the angiogenesis progression and observed that it could inhibit cell proliferation, capillary-like structure formation and motility.

### Glioblastoma

Glioblastoma is the most common primary brain tumor in adults and usually leads to rapid death. A therapeutic option considers targeting the death receptor (DR) to activate tumor cell death pathway but the variable response degree in tumor cells to DR agonist-mediated apoptosis represents a major limitation of this approach.

A bispecific construct (ENb-TRAIL) composed by an anti-EGFR nanobody and the DR ligand TRAIL demonstrated therapeutic efficacy in tumor cells that do not respond to either EGFR antagonist or DR agonist monotherapies (**Figure 1**). ENb-TRAIL induced a triple anti-tumor effect by inducing DR5 aggregation in the plasma membrane, by initiating caspase-mediated apoptosis of tumor cells and by blocking the EGFR signaling pathway. *In vivo* assays proved that ENb-TRAIL treatment significantly alleviated tumor burden and increased survival (20, 68). A tetravalent anti-DR5 agonistic nanobody construct (TAS266) showed more potency than the ENb-TRAIL compound in pre-clinical studies but did not passed Phase I due to its strong hepatotoxicity (69).

Brain is also the site of metastatic tumors. A bispecific anti-VEGF-A/Ang2 nanobody was able to reduce significantly number and volume of metastatic tumors in a mouse model (70).

### Lung Cancer

Lung cancer is the leading cause of cancer death worldwide and, among the different subtypes, non-small cell lung cancer (NSCLC) accounts for about 85% of the whole cases (71). The trifunctional bispecific antibody Catumaxomab based on Quadroma (Hybrid Hybridoma) technology, which combines the EpCAM rat antibody (IgG2b) and CD3 mouse antibody (IgG2a) into a bispecific molecule (56), was the first multispecific antibody approved by EMA (2009) for the treatment of NSCLC malignant ascites (**Figure 2**). Other not exclusive biomarkers, such epidermal growth factor receptor (EGFR), are often considered as therapeutic targets in NSCLC because highly overexpressed and often mutated. An anti-EGFR nanobody combined with photosensitizer and catalase succeeded in improving tumor hypoxia and consequently killed A549 primary tumor and inhibited lung metastasis, prolonging mouse survival (72). Also anti-EGFR nanobodies functionalized with a cell-penetration poly-arginine peptide were highly cytotoxic to the same human adenocarcinomic alveolar basal epithelial cells A549 (73).

About 33% of patients with NSCLC tumors and epidermal growth factor receptor mutations develop brain metastasis (74). Osimertinib is effective against mutated EGFR but drug resistance raises in 1 to 2 years (75). To overcome this limitation, dual-targeting liposomes were generated that display an anti-PD-L1 nanobody and a transferrin receptor-binding peptide. This construct is still able to pass the blood-brain barrier and mediate simvastatin/gefitinib delivery to the NSCLC-EGFR^mut^ metastatic tumor (76).

Nanobodies were also exploited to assess alternative biomarkers and therapeutic options. A technetium-99 m-labeled anti-CEA nanobody was used to prove that the carcinoembryonic antigen (CEA) was a potentially interesting marker for NSCLC (77), whereas bright nanoprobes based on quantum dots conjugated to anti-HER2 nanobodies provided better immunolabeling of lung cancer cell lines than dyes Alexa Fluor 488 and Alexa Fluor 568 (78). Multivalent anti-CCR8 nanobodies fused to Fc were also effective in NSCLC treatment by eliciting antitumor immunity through tumor-promoting Treg cells depletion and ADCC activation (79). Since angiogenesis is critical in tumor development, VEGFR2 was targeted by an anti-VEGFR2 nanobody conjugated with the enzyme urease that can convert endogenous urea into ammonia, a product toxic to tumor cells (80).

### Breast Cancer

Breast cancer is the second most common cancer in women and the one causing more deaths. According to the genetic and biochemical characteristics, breast tumors are grouped into three major classes (Estrogen/Progesterone receptor positive, Her2 positive, Triple Negative - TN), with further stratification describing the subtypes of Triple Negative (81). About 20% of breast cancers are characterized by amplification of the HER-2/neu gene (82). HER2 is a tyrosine kinase receptor the overexpression of which causes increased tumor cell proliferation, tumor invasiveness, accelerated angiogenesis, and reduced apoptosis (83). Anti-HER2 monoclonal IgG antibodies trastuzumab (Herceptin) and pertuzumab, which target independent epitopes, are widely used alone or in combination in clinical treatment of HER2-positive breast cancers (84, 85). Nanobodies that bind to the same epitopes recognized by such IgGs have a potential therapeutic interest (36, 42, 86), whereas those that do not interfere with their binding because target further epitopes are suited for imaging or can be used as complementary theranostic reagents (87–90).

Since nanobodies specific for Her2 are simple to generate (43) and as recombinant proteins are simple to engineer, anti-Her2 nanobodies have been exploited to develop an enormous variety of reagents, such as bivalent and biparatopic molecules (25), immunotoxins (91, 92), activated nanoparticles (25, 93–95), biosensor immunocapture surfaces and other nanostructures suitable for receptor detection (96–101) and have been also *in silico* modeled to increase their biophysical features (102). As a general rule, multivalent formats provide higher apparent affinity for their antigen due to the avidity effect. Furthermore, multispecific formats are often more effective in inhibiting cell proliferation because of their capacity to block contemporarily alternative activation pathways (19, 103). The number of nanobodies available for TN breast cancers is extremely lower. TN positive cells have been successfully targeted with anti-EGFR-activated Quantum-Dot theranostic micelles, anti-STAT3 and anti-Protein C Receptor nanobodies and anti-TNFα nanobodies used alone or fused to a recognition peptide binding to the αvβ3 receptor on tumor cell membranes (104–108).The common limit of these approaches is that the targeted biomarkers are not exclusive of TN cells and therefore their clinical usefulness remains to be demonstrated. There is no report of nanobodies used for the diagnostic or the therapy of Estrogen/Progesterone receptor positive breast tumors.

### Ovarian Cancer

Ovarian cancer is characterized by tumor heterogeneity and by controversial diagnostic methodology (109). Nanobody-activated nanospheres were successfully used for developing a highly sensitive (detection limit of 0.560 pg mL^-1^) photoelectrochemical biosensor able to detect the serum biomarker Human Epididymis Protein 4 (HE4) and clearly distinguish ovarian cancer patients from healthy individuals (110). Mucins are type I membrane O-glycoproteins with single transmembrane domains that are usually highly upregulated during tumorigenesis and could represent a therapeutic target (111). In this perspective, Li et al. (27) designed a bispecific construct (Muc1-Bi) constituted by two nanobodies, one specific for Muc1 and the second for CD16 that could be applied for ovarian cancer treatment. This reagent can recruit NK cells and drive them to Muc1-overexpressing tumor cells and, in a xenograft model, significantly suppressed tumor growth.

### Colon Carcinoma

Despite being expressed in several tumors, CEA is often used as a biomarker of colon carcinoma (112). CdSe/ZnS quantum dots conjugated to an anti CEA nanobody have been used as efficient two-photon excitation probes for imaging colon carcinoma tissue (113), whereas a bispecific construct formed by linking anti-CEA and anti-CD16a nanobodies succeeded in engaging NK cells and inhibiting CEA-overexpressing tumor growth *in vivo* (30) (**Figure 1**). A novel bispecific nanobody with dual PD-L1/TIGIT demonstrated high inhibitory activity towards both PD-1/PD-L1 and TIGIT/CD155 interactions. Its application synergistically enhanced T cell activity *in vitro* compared to that of the two parental nanobodies (38) and such strategy for treating tumors might improve the reliability of therapies aiming at immune checkpoint blockade.

## Nanobody Applications for Autoimmune Diseases

Antibodies represent a powerful means for the treatment of immune diseases because they can target ligands or receptors involved in the abnormal amplification of molecular signals responsible for the symptoms. Currently, there are antibodies approved for treating autoimmune diseases such as rheumatoid arthritis, inflammatory bowel disease, type 1 diabetes, psoriasis, lupus, and multiple sclerosis (114). Even though this implies the necessity of more frequent treatments, antibody fragments with short half-life period are considered safer because of their reduced retention time and their engineering into dual-target reagents provides the advantage of blocking pairs of inflammatory cytokines at the same time, increasing the treatment efficacy (7) (**Table 3**).

**Table 3:** Multivalent mono/bispecific nanobodies for autoimmune diseases.

Nanobody	Disease	Target	Structure features	Year	Reference
MT1 – MT1	Inflammatory Bowel disease	TNF-α	Bivalent monospecific	2010	(115)
ATN-103	Rheumatoid artheiris	TNF-α	Bivalent monospecific	2012	(116)
TROS	Inflammatory Bowel disease	TNFR1	Bivalent bispecific	2015	(117)
37D5-Alb1-124C4	Chronic inflammation	IL23	Bivalent monospecific	2017	(118)
VHH#3-9GS-VHH#1	Inflammatory Bowel disease	TNF-α	Bivalent monospecific	2017	(119)
M1095	Psoriasis	IL-17A/F	Bivalent bispecific	2017	(120)

### Inflammatory Bowel Disease

Inflammatory bowel disease (IBD) is a multifactorial disorder characterized by chronic and relapsing intestinal inflammation. IBD includes ulcerative colitis (UC) and Crohn’s disease (CD) that affects the ileum, rectum, and colon. Nowadays, inflammatory bowel disease cannot be completely cured, but anti-TNF-α monoclonal antibodies, such as infliximab and adalimumab, have made a significant breakthrough by enabling the delay of IBD progression (121). Moreover, their rapid mucosal healing ability has improved the response and remission rate of patients with IBD, especially for CD treatment (122). Tumor necrosis factor (TNF) is a pro-inflammatory cytokine that represents a critical mediator of the autoimmune process, playing a key role in several inflammatory diseases, including rheumatoid arthritis (RA), ulcerative colitis, and CD. An innovative therapeutic approach for treatment of chronic colitis considered the *in situ* secretion of anti-TNF nanobodies by orally administered *L. lactis* bacteria engineered to secrete monovalent and bivalent anti-TNF nanobodies that neutralized TNF *in vivo* (115). TNF is the target of further inhibitory nanobodies. A trispecific anti-TNF construct could effectively inhibit the TNF/TNFR1 signaling pathway and its inhibitory activity was successfully tested *ex vivo* using colon biopsies of CD patients (117). In another study, three anti-TNF nanobodies with sub-nanomolar affinity for their antigen were selected and the crystal structures of the TNF–nanobody complexes showed that they targeted (partially) overlapping epitopes (119). Nevertheless, bivalent molecules showed increased blocking activity due to the fact that, differently from conventional antibodies, these constructs can bind simultaneously to two independent receptor binding sites of the trimeric TNF.

Recent studies have shown that IL-23 mediates the over-proliferation of T(H)-17 cells and the resulting accumulation of IL-17 and IL-22 pro-inflammatory cytokines promotes dermal inflammation and CD pathogenesis (123). Anti-human IL-23 nanobodies with low nanomolar affinity for hIL-23 and targeting independent epitopes were assembled together with an anti-human serum albumin nanobody into multivalent constructs. They showed prolonged *in vivo* half-life and improved hIL-23 neutralization capacity *in vitro* and *in vivo* with respect to the single monomeric nanobodies (118).

### Psoriasis

The above described anti-IL23 multivalent nanobodies might be suitable for treating psoriasis as well. Furthermore, current studies suggest that another effective treatment method would be the inhibition of IL-17 (120). M1095 is a trivalent bispecific-nanobody that can effectively neutralize the pro-inflammatory cytokines IL-17A and IL-17F as well as bind to human serum albumin. The initial clinical trials to evaluate the safety and effectiveness of the immunoreagent in patients with moderate to severe psoriasis showed that M1095 was well tolerated whereas psoriasis-related inflammatory markers were significantly decreased (120).

### Rheumatoid Arthritis

Rheumatoid Arthritis (RA) is a common chronic autoimmune disease. Tumor necrosis factor-α (TNF-α), as a pleiotropic cytokine, induces adverse pro-inflammatory and cytotoxic effects in the course of RA. A preliminary work showed that antagonistic anti-TNF nanobodies linked to an anti-serum albumin nanobody were 500 times more effective than monovalent nanobodies in controlling rheumatoid arthritis development in a mouse model (124). The bispecific nanobody format showed also higher antagonistic potency than the commercial IgG antibodies infliximab and adalimumab. On the base of such experience, it was designed the compound ATN-103 (ozoralizumab), a trivalent bispecific albumin-conjugated nanobody that targets TNF-α. In Phase II clinical trials it showed to cause no immunogenic response (116). Taisho Pharmaceuticals performed an apparently successful Phase III with this multimeric nanobody and recently submitted an application for approval to manufacture and market the immunodrug (**Table 1**).

Since also interleukin 6 plays a key role in the pathogenesis of RA, such cytokine was targeted by the bispecific nanobody ALX-0061 that binds as well to human serum albumin, recruited to extend the construct half-life. In cynomolgus monkeys, ALX-0061 induced a dose-dependent inhibition of IL-6-induced inflammatory parameters (125).

## Nanobody Applications in Infectious Diseases

The development of new vaccines and of antibiotics effective on multi-resistant bacteria is difficult and time-consuming. In recent years, nanobodies with neutralizing toxin activity have been studied for the treatment of bacterial toxins, such as those produced by *Clostridium difficile*, *Bacillus anthracis*, ricin and anthrax. These accomplishments show that nanobodies represent an alternative anti-infection therapeutic opportunity against bacterial and viral outbreaks (126, 127) (**Table 4**).

**Table 4:** Multivalent/bispecific nanobodies for infectious diseases.

Nanobody	Disease	Target	Structure features	Year	Reference
D3n(GS)2	Respiratory	Fusion protein	Bivalent	2011	(128)
D3/E4	Syncytial Virus		Biparatopic		
C12 n(GS)2	Rabies virus	Glycoprotein	Bivalent	2011	(128)
E8/H7			Biparatopic		
C8 n(GS)2	H5N1 Influenza	Hemagglutinin 5	Bivalent/trivalent	2011	(128)
ARP1–ARP1	Rotavirus	RRV	Bivalent	2011	(129)
ARP3–ARP1			Bispecific		
T5-V36	Tetanus Toxin	TerC/Mac-1	Bispecific	2015	(130)
JJX12	Ricin Toxin	RTA/RTB	Bispecific	2016	(131)
Ad/	*Bacillus anthracis*	Lethal factor/edema factor	Bispecific	2016	(132)
VNA2-PA	Lethal/edema toxin				
Liposomal Vhhs	HIV	gp120	Multivalent monospecific	2016	(133)
VUN401-Fc	HIV	CXCR4	Bivalent monospecific	2018	(134)
Nb70-alb-14	Acute Inflammation and Sepsis	TNFR1/MMP8	Bivalent bispecific	2018	(135)
NbF12-10	*Androctonus australis hector* scorpion venom toxins	AahI/AahII	Bispecific	2018	(136)
V _H_ H-V _H_ H dimers	*Clostridium difficile* toxin B	CROPs domain	Bivalent	2018	(137)
Nb113_2_	*Escherichia coli* Shiga toxin	Stx2a	Bivalent	2018	(138)
J3-2E7	HIV	gp41/gp120	Bivalent bispecific	2019	(139)
Nb 2TCE49	Human toxocariasis	TES	Bivalent	2019	(140)
F1×F1-hFc	Hand, foot, and mouth disease (HFMD)	Enterovirus A71	Tetravalent	2020	(141)
H11-D4-Fc	COVID-19	SARS-CoV-2 spike RBD	Bivalent	2020	(142)
H11-H4-Fc					
Nb-Fc	COVID-19		Multivalent	2020	(143)
Cocktail nanobody	COVID-19		Multivalent	2020	(144)
			Multi-epitope cocktail		
Nbs 20/21	COVID-19	SARS-CoV-2 spike RBD	Trivalent	2020	(145)
sACE2-anti-CD16 VHH	COVID-19	RBD/CD16	bispecific	2021	(146)
Nb15-NbH-Nb15	COVID-19	SARS-CoV-2 spike glycoprotein/HSA	Trivalent bispecific	2021	(147)
hIgG1Fc-VHH	Bunyaviruses	RVFV/SBV	Tetravalent bispecific	2021	(148)
aRBD-2-5; aRBD-2-7	COVID-19	RBD	Bispecific	2021	(149)

### Acute Inflammation and Sepsis

The lack of specific treatment for sepsis leads to the worldwide incidence of 31.5 million of cases and to 5.3 million deaths per year. Sepsis severity is associated with plasma levels of matrix metalloproteinase-8 (MMP8) and tumor necrosis factor receptor (TNFR1) and, therefore, the effect of a bispecific nanobody able to block simultaneously MMP8 and HTNFR1 was evaluated (135). The results obtained in mouse model indicated that the nanobody-dependent neutralization of MMP8 and HTNFR1 had a beneficial effect in terms of survival rate. Different combinations of biparatopic nanobodies conferred 100% survival upon prophylactic or up to 24 hour post-infection administration in pneumonia mouse models challenged with *Pseudomonas aeruginosa* (150).

### Viral Infection

Viral infectious diseases have been one of the leading killers in the history of mankind. Influenza A virus is the main pathogen causing human influenza, it can infect a variety of animals and cause cross-species infection (151). Influenza virus neuraminidase (NA) plays an important role in the release and spread of the virus as well as in the cellular infection and consequently is a potentially interesting therapeutic target (152). Cardoso et al. (2014) isolated a set of anti-H5N1 NA nanobodies and generated bivalent molecules either connecting two single-domains with a flexible linker or by exploiting the dimerization properties of a mouse IgG2a-Fc domain fused to each single domain (153). The results showed that bivalent nanobodies had an *in vitro* antiviral potency 30- to 240-fold higher than monovalent nanobodies and protected BALB/c mice from H5N1 infections when used as a prophylactic therapy. Multimeric constructs were also conceived to protect simultaneously from different viruses. Specifically, Hultberg et al. (2011) prepared a trimeric construct linking nanobodies specific for H5N1 Influenza (able to neutralize both clade1 and 2), Respiratory Syncytial Virus and Rabies virus that protected from any of the three viruses (128). The authors also demonstrated that playing on the format (different bivalent and biparatopic combinations of single nanobodies), it was possible to increase the potency of the neutralizing anti-viral reagents and concluded that multimerization of nanobody fragments targeting multiple epitopes on viral trimeric spike proteins is a powerful tool for anti-viral therapy with broader neutralization capacity. The trivalent nanobody construct ALX-0171, designed for the inhalation treatment of respiratory syncytial virus infection, had a remarkable capacity to reduce escape mutant selection and showed promising results in animal models (154, 155) but was finally unable to improve clinical course in patients with established infection in the lower respiratory tract (156). Based on the results of such Phase IIb dose-ranging study, the Sponsor decided to discontinue ALX-0171 trials.

Enterovirus A71 (EVA71) is a major cause of viral encephalitis and severe hand, foot, and mouth disease (HFMD) in young children worldwide. Neither preventive not therapeutic treatments are available for limiting EVA71 infection. Huang et al. (2020) isolated a nanobody (F1), which inhibited EVA71 infection both *in vitro* and *in vivo*. The neutralizing activity was improved when multivalent formats were used and the most effective constructs were the ones in which the structure geometry enabled to maximally exploit the avidity effect (141).

Gastroenteritis induced by rotavirus infection is a major health problem in development countries. Bispecific nanobody constructs targeting independent rotavirus epitopes were used to transform *Lactobacilli.* Next, *Lactobacilli* cultures were used to deliver the neutralizing nanobodies to the intestinal lumen and such posology showed high anti-virus efficiency (129).

### HIV-Dependent Immunodeficiency Syndrome

Acquired immunodeficiency syndrome (AIDS) causes about 1.8 million AIDS-related deaths each year (157) and is caused by infection with HIV (Human Immunodeficiency Virus). HIV leads to extensive destruction of T-helper cells, macrophages, dendritic cells, and other cellular components associated with cell-mediated immunity, eventually leading to the destruction of the immune system. Consequently, the organism becomes the target of many opportunistic diseases (158). At the present, the effective antiretroviral therapy (HAART) renders HIV a chronic disease (159) but this treatment is expensive and has many adverse effects (160). Therefore, there is still an urgent demand for effective and low-cost treatment of HIV infection.

Experiments made with combinations of patient-derived HIV neutralizing antibodies targeting complementary epitopes demonstrated that the inhibition effect was increased when more virus epitopes were blocked contemporarily (161). A contribution to the effort of isolating antibodies with complementary characteristics was the method described by Koh et al. that allowed the recovery of nanobodies with distinct binding features (162). However, no further studies with multispecific constructs built using the selected binders were published, despite the encouraging results showing that nanobody homo- or heteromultimers could neutralize a wide array of virus subtype (163). A bivalent nanobody targeting the proximal external region of gp120 had a neutralization capacity 20 times higher than the monovalent ligand (164) and a bispecific construct targeting gp41 and gp120 epitopes possessed a neutralizing potency up to 1400-fold higher than the mixture of the individual nanobodies (139). Liposomes displaying nanobodies were also proposed to increase avidity, but the results were deceiving because the low nanobody density impaired the simultaneous binding to more than one target protein (133). More promising it seems the alternative of using neutralizing nanobodies displayed on the surface of *Lactobacillus rhamnosus* cells (165).

An alternative approach considered targeting the CXCR4 receptor that participates in the viral uptake (134). Bivalent constructs were constructed by linking nanobodies to a human IgG1 antibody Fc domain and were compared with their monovalent counterparts. The Nb-Fc constructs had higher binding affinity, blocked more efficiently the CXCR4-mediated HIV entry and induced ADCC- and CDC-mediated cell-death of CXCR4-overexpressing cells, a condition that renders such molecules attractive to treat also CXCR4-overexpressing tumors.

### SARS-CoV-2 Infection

SARS-CoV-2 is a single-stranded RNA virus that belongs to the CoVs family. The receptor-binding domain (RBD) of the spike protein binds to the host cell surface receptor angiotensin-converting enzyme 2 (ACE2). Several anti-spike/anti-RBD neutralizing antibodies have been isolated from both patients and by *in vitro* selection, some of them have entered clinical trials and cocktails of ligands binding to different epitopes have been proposed to overcome resistance due to the virus mutations (127, 166). Multispecific antibody fragments are an alternative solution to prevent mutation-dependent resistance, as it has been already summarized in recent reviews (34, 127, 167). Since the research on anti-SARS-CoV-2 is particularly active and new publications appear constantly, here we report only those relevant to the subject of the present review and published in the first 2021 quarter.

Heterodimers formed by nanobodies recognizing independent epitopes and connected by fusion with the human Fc fragment exhibited the strongest RBD-binding affinity and neutralizing ability against SARS-CoV-2 pseudoviruses (143). In this case, the nanobodies were isolated from a naïve library but the same strategy of using nanobody-based Fc-dependent heterodimers to increase both the apparent affinity and the neutralizing activity of the immune reagents was successfully exploited by another group that used immunized alpaca as the source of the ligands (149). The recognized advantage of targeting independent epitopes was also used to change paradigm in the preparation of multivalent constructs: instead of assembling randomly isolated, not overlapping nanobodies, these were chosen according to the available structural information (168). This strategy enabled to design biparatopic constructs with apparent affinity in the pM range starting from nanobodies with 10-100 times lower affinities (169, 170).

The other actual acute problem posed by the pandemic is the emergency of virus variants. Specifically targeting the single mutations might be extremely demanding, but a recent work (171) demonstrated that it is possible to have a large array of neutralizing nanobodies specific for several independent epitopes of the conserved regions, the combined use of which should offer protection options even in the case of highly mutated virus forms.

### Anti-Toxin Nanobodies

In the case of toxin infection, nanobodies have the pivotal advantage over IgG that most of them can be expressed as functional intrabodies and therefore can be directly produced inside host mammalian cells as a protective antidote. The toxin neutralizing capacity of a prophylactic gene therapy has been successfully demonstrated in the case of *Bacillus anthracis* infection, the causative agent of anthrax (132). The protective antigen (PA) is the common component of *B. anthracis* toxins. The sequences of two anti-PA nanobodies targeting two independent antigen epitopes were cloned in an adenovirus vector to produce a bispecific immunoreagent. Mice were injected with the vector and the resulting nanobody construct that accumulated in their sera protected the animals by infections with anthrax toxins and spores.

*Clostridium difficile* is a problematic nosocomial pathogen that can cause diarrhea, pseudomembrane colitis and even death due to the effect of the virulence factors TcdA and TcdB toxins (172). Monomeric nanobodies targeting different TcdB epitopes were incapable of preventing TcdB-induced cytotoxicity in cell-based assays, despite their very high-affinity. However, the toxic effect inhibition was achieved when nanobodies were prepared into Fc-dependent bivalent constructs (137).

Shiga toxin-producing *Escherichia coli* (STEC) are a subset of potentially lethal pathogens. Tandem repeats of nanobodies targeting the Shiga toxin-2a B subunit provided toxin neutralization capacity 100 times higher than the monovalent constructs (138). For the neutralization of tetanus neurotoxins, the best results were obtained by fusing an anti-toxin nanobody to another specific for Mac-1, a surface integrin receptor expressed on most innate immune cells that plays an essential role in the elimination of complement opsonized microorganisms (130). The bispecific construct allowed mice to survive a 10-fold lethal dose with respect to monomeric anti-toxin and outperformed a sheep anti-toxin polyclonal IgG. Another bispecific nanobody construct alleviated ricin toxin effects by promoting its aggregation and by modifying the dynamics of ricin uptake and its cellular trafficking (131).

Multivalent/multispecific nanobody constructs were also beneficially used for diagnostic goals. Bispecific nanobodies were used to construct an electrochemical biosensor to detect the scorpion venom toxins AahI and AahII (136). In the case of human toxocariasis, the most effective ELISA test was obtained using bivalent nanobodies for antigen capture (140). The *Alternaria* mycotoxin tenuazonic acid was conveniently detected with high sensitivity using a one-step bioluminescent enzyme immunoassay that required the bifunctional fusion formed by nanobody and nanoluciferase (173).

## Nanobodies for Further Therapeutic Applications

Despite most of the applications for nanobody-based reagents were conceived for few specific research areas, their potential usefulness appears confirmed also in further fields (**Table 5**).

**Table 5:** Other multivalent/bispecific nanobodies with therapeutic potential.

Nanobody	Disease	Target	Structure features	Year	Reference
FAF-Nb	Gelsolin amyloidosis	D187N/Y gelsolin/HSA	Bivalent	2014	(174)
			Bispecific		
Nb22-FAF-Nb	Gelsolin amyloidosis	C68/amyloidogenic gelsolin-fragment	Bivalent	2017	(175)
			Bispecific		
Everestmab	Type 2 diabetes mellitus	GLP-1R/HSA	Bivalent	2020	(176)
			Bispecific		
VHH-B11	Cardiovascular diseases	Low density lipoprotein cholesterol	Bivalent	2020	(177)
BI-X	Retinal vascular diseases	VEGF/Ang-2/HSA	Multivalent	2021	(178)
			Multi-specific		

### Type 2 Diabetes Mellitus

Type 2 diabetes mellitus is a chronic metabolic disorder disease characterized by hyperglycemia and associated comorbidities (179). Glucagon-like peptide-1 (GLP-1) plays an essential role in glucose homeostasis by binding to and activating the GLP-1 receptor but its extremely short half-life (3 min) limits the effect its therapeutic administration. Everestmab is a tri-functional construct developed to overcome this drawback. It is a fusion protein consisting of a mutated GLP-1, necessary for the biological activation of the GLP-1 receptor, an anti-GLP-1 nanobody suitable for the construct targeted delivery, and an anti-HSA nanobody to prolong its circulation time of the construct to several days. Everestmab treatments produced promising results in animal models (176).

### Retinal Neovascular Diseases

Neovascular age-related macular degeneration and diabetic retinopathy are major causes of visual impairment and blindness, requiring frequent intravitreal injections of anti-angiogenesis biotherapeutics (180). The trispecific nanobody construct BI-X was designed for targeting simultaneously both the angiogenesis factors VEGF-A and Ang-2 and human albumin to increase the molecule half-life (178). BI-X showed superior efficacy and significant half-life extension when tested in cynomolgus monkeys after intravitreal injection. However, intravitreal injection is a burden for patients and has potential complications, such as infections and topic applications will be tested.

### Neurodegenerative Diseases

Nanobodies have been highly used to study the structure and the development of protein aggregates involved in the progression of neurodegenerative diseases (52, 181–183). A recent review illustrates how multimeric constructs based on antibody fragments can be used as effective intrabodies (184) for re-targeting antigen-antibody complexes. Specifically, nanobodies were engineered to favor PEST-dependent degradation of α-Synuclein (α-Syn) (185, 186). Nanobody bioconjugates were also successfully used as imaging probes able to cross the blood brain barrier and label amyloid-beta deposits after intravenous injection (187) as well as to functionalize gadolinium-based nanoparticles that allowed the visualization of amyloid fibril deposits in pathological tissues (188). Anti-α-Syn nanobodies fused to a fluorescent probe enabled to monitor the cytosolic presence of the antigen and to reveal the presence of transmittable αSyn in human cerebrospinal fluid (189).

Gelsolin amyloidosis, also known as familial amyloidosis of the Finnish type, is an autosomal dominantly inherited systemic disorder with ophthalmologic, neurologic, and dermatologic symptoms (190). A single point mutation (D187N) results in a pathological proteolytic cascade with the formation of amyloidogenic peptides which aggregate in multiple tissues and cause disease-associated symptoms. Nanobodies exclusively selective for one of the amyloidogenic fragment, but not for the wild type protein, acted as molecular chaperones and mitigated the aggregation process (174). Their effect *in vivo* was significantly improved when the animals were treated with a bispecific molecule obtained by coupling the anti-gelsolin nanobody with an anti-albumin nanobody. The same group demonstrated the protective effect of such construct when it was directly expressed by mutant mice that underwent adenovirus-based gene therapy (175).

### Others

A nanobody inhibiting the proprotein convertase subtilisin/kexin type 9 (PCSK9) was expressed fused to an Fc domain and effectively reduced the production of low density lipoprotein (LDL) and cholesterol in a rat model (177). The results were comparable to those obtained with the approved monoclonal evolocumab but at extremely lower production costs.

The bispecific nanobody ALX-0962 that targets both IgE and human serum albumin can neutralize soluble IgE as well as displace preformed IgE-FcϵRI complexes and therefore was considered for the treatment of allergic asthma (191).

## Discussion

Since Nisonoff and his colleagues proposed the concept of bispecific antibody 60 years ago, therapeutic bsAbs have made great progresses (192). At the present, over 85 bsAbs are progressing through clinical development for a wide variety of indications (193). The most attractive feature of BsAbs is that they have features and provide effects that are not present in a simple combination of single antibodies, such as increased avidity and selectivity. Furthermore, by targeting multiple molecular targets simultaneously, multi-specific antibodies can block independent signal pathways, a condition that contributes effectively to prevent drug resistance and immune escape. For instance, targeting simultaneously EGFR and VEGFR2 by means of a bispecific construct composed by fragments of the parental IgGs cetuximab and ramucirumab was effective in inhibiting EGFR-dependent tumor growth and VEGFR2 angiogenic pathway in a mouse model of Triple Negative Breast Cancer (194), whereas a bispecific antibodies targeting TGF-β and PD-L1 showed a superior anti-tumor effect with respect to monotherapy due to enhanced anti-tumor immune response in multiple *in vivo* models (195).

**Figure 3:**
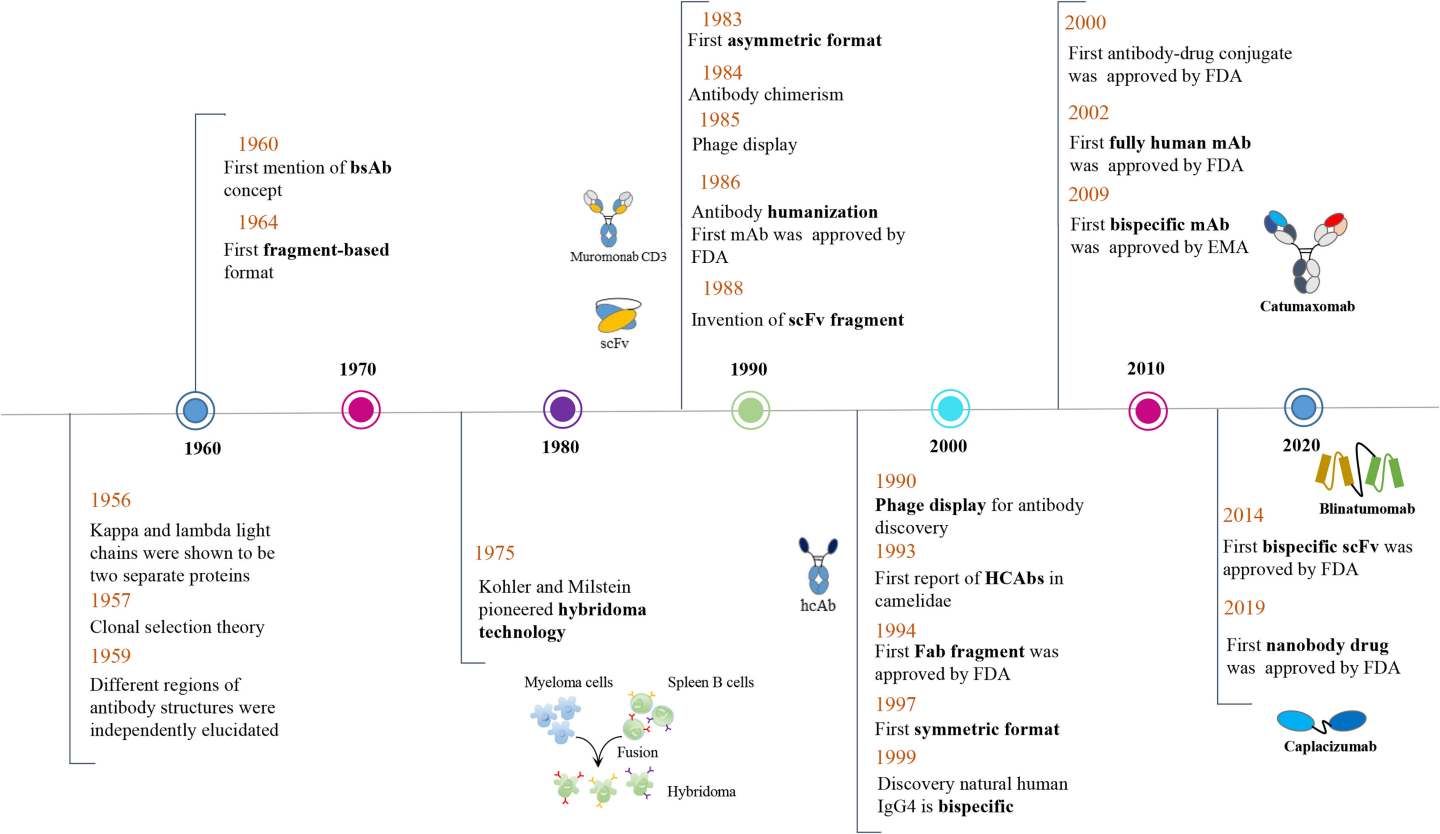
Overview of the publications dedicated to BsNbs. Number of published papers reported in PubMed dedicated to studies describing the use of multispecific/multivalent nanobodies and grouped according to the addressed pathology.

Nowadays, nanobodies are regarded as an alternative to monoclonal antibodies because they overcome some of the IgG drawbacks, such as their low penetration in solid tumors and tissues, the difficulty to control their functionalization process and their elevated production costs in mammalian cells (15). Nanobodies small dimension allows deeperpenetration in solid tumors with respect to IgGs the effect of which is usually limited to the superficial cells. On the other hand, their limited mass favors also a rapid kidney filtration with consequent very short circulation half-life. This represents an advantage for *in vivo* imaging because rapidly reduces the background signal but it is the major shortcoming for therapeutic applications. However, the recombinant nature of nanobodies can be exploited to produce fusions with relevant proteins and tags that possess reduced clearance and even additional useful functions such as fluorescence or cytotoxicity or enable the building of multivalent and multi-specific constructs with largely diversified formats (10, 196–198). Multi-specific nanobodies retain at least part of the advantages of nanobodies, providing a mass that is still significantly smaller than IgGs and the capacity of targeting hidden epitopes by means of their protruding paratopes. Furthermore, their *in vivo* half-life can be fine-tuned according to the application needs (199). It must be also considered that nanobody biophysical features allow their administration *via* delivery routes that are not accessible to conventional IgGs. In addition to subcutaneous and intravenous injections, nanobodies can be nebulized directly into the respiratory tract (156), taken orally for gut treatment (165) and topically for ophthalmic applications. The PubMed data indicated that sixty research papers/year dealing with multi-valent/multi-specific nanobodies, half of them dedicated to cancer research, were published in the last two years (**Figure 3**). The research output indicates that multivalent and multi-specific constructs are more effective that their corresponding monomers. This evidence was confirmed any time that the monovalent and multivalent formats were compared, with no exception and independently on the strategies used to obtain multivalent structures. Also, nanobody derivatization with enzymes, dyes, chelators and other functional tags resulted in reliable and effective immunoreagents. In this case, the decisive advantage with respect to IgG was that the derivatization process was controlled, namely 1:1 and at a specific residue. This condition avoids the generation of heterogeneous populations of reagents characterized by different number of added functions at different residues, as detected when lysine amino groups are used (196).

Nanobodies are not particularly immunogenic and different humanization strategies have been conceived (86, 200) and such condition might simplify the clinical trials of nanobody-derived medicaments. Caplacizumab was the first nanobody-based approved drug (58) but despite several multivalent or multi-specific nanobodies entered clinical trials, so far none has been approved (201). Companies usually do not divulgate the reasons of clinical trial failures but we can argue that these are the result of several factors. The delivery kinetic of such molecules can be affected by single patient proteome profile and they can show higher immunogenicity due to the creation of new epitopes corresponding to the linking sequences. Furthermore, such complex molecular structures require the precise three-dimensional positioning of the single domains to achieve cooperative interaction with the antigen but so far there is no single evidence that a bispecific antibody could simultaneously bind to both epitopes *in vivo*. More probably, the generally observed higher efficacy of bispecific antibodies over treatments with monospecific antibodies or combination of them is due to an avidity effect allowed by the co-presence of both target epitopes in the same environment. Therefore, it will be necessary to further improve the structure design of multidomain binders by calculating the optimal distance between the paratopes in a way that enables the simultaneous binding to their corresponding epitopes. The research aimed at the high-resolution mapping of the receptors on the cell surface has provided meaningful information relatively to cluster density and composition (93, 202). These data are the base on which the next generation of multispecific antibodies will be probably designed. In this perspective, nanobody short sequence represents a decisive advantage because the computing requirements for their modeling are extremely less expensive than those necessary for larger binders. Computer-aided nanobody design technology has strongly improved in the last few years (86, 203–208) and will be more and more reliable for providing solutions also to these challenges.

Altogether, the accumulated experimental data and the available tools suggest that multimeric and functionalized molecules built using nanobodies will become a major component of future diagnostic and therapeutic reagents.

## Data Availability Statement

The original contributions presented in the study are included in the article/supplementary material. Further inquiries can be directed to the corresponding authors.

## Author Contributions

HH, AM, JW, and GK contributed to the conception of this review. JW and GK wrote the first draft of the manuscript. HY is responsible for literature retrieval. HH, AM, JW, GK, and HY wrote sections of the manuscript. AM and HH supervise the project administration. All authors contributed to the article and approved the submitted version.

## Funding

The present study was supported by grants from the National Key Research and Development Project (Grant No. 2019YFA0905600); the Science and Technology Program of Tianjin, China (Grant No. 19YFSLQY00110), the Major State Basic Research Development Program of the Natural Science Foundation of Shandong Province in China (Grant No. ZR2020ZD11), and by the grant ARRS/PA-0107 provided by the Javna Agencija za Raziskovalno Dejavnost Republike Slovenije. We thank Shaoxing “MingShiZhiXiang” Meritocrat Project and Program of Introducing Talents of Discipline to University Ministry of Education, China - 111 Project (Grant No. BP0618007) for its support.

## Conflict of Interest

The authors declare that the research was conducted in the absence of any commercial or financial relationships that could be construed as a potential conflict of interest.

## Publisher’s Note

All claims expressed in this article are solely those of the authors and do not necessarily represent those of their affiliated organizations, or those of the publisher, the editors and the reviewers. Any product that may be evaluated in this article, or claim that may be made by its manufacturer, is not guaranteed or endorsed by the publisher.
